# Meta-modeling the effects of anodal left prefrontal transcranial direct current stimulation on working memory performance

**DOI:** 10.1162/imag_a_00078

**Published:** 2024-01-25

**Authors:** Miles Wischnewski, Taylor A. Berger, Alexander Opitz

**Affiliations:** Department of Biomedical Engineering, University of Minnesota, Minneapolis, MN, United States

**Keywords:** working memory, transcranial direct current stimulation, electric field modeling, prefrontal cortex, neuromodulation

## Abstract

Numerous studies have demonstrated the beneficial effects of anodal prefrontal transcranial direct current stimulation (tDCS) on working memory. However, a large variability exists in the applied tDCS parameters and working memory outcome measures. Using a meta-modeling approach, we investigated the relationship between tDCS electric fields in the left prefrontal cortex and improvements in working memory performance. Using this approach, a vector of outcome measures is correlated with the tDCS-related electric fields across several studies. These performance-electric field correlations (PEC) are calculated for each spatial location of the grey matter. Extracting 354 data points from 67 studies, we compared the spatial maps of tDCS effects on I) working memory accuracy and speed (regardless of working memory type and time of assessment), II) verbal and visuospatial working memory (regardless of performance measurement and time of assessment), and III) performance during and after stimulation (regardless of performance measurement and working memory type). We found that accuracy improves when anodal tDCS is applied to inferior frontal regions (Brodmann area 47) while working memory speed benefits from stimulation to dorsolateral and anterior prefrontal areas (Brodmann areas 9/10). Furthermore, the beneficial effects of left prefrontal tDCS are exclusive to verbal working memory, with no improvements in visuospatial working memory. We also observed region-specific effects only for task performance during, but not after, stimulation. The results of this study elucidate the causal involvement of prefrontal regions in working memory and can help guide tDCS placement for therapeutic application in disorders that involve working memory deficits.

## 1 Introduction

Working memory, which refers to the temporary storage and manipulation of information, is necessary for a large variety of complex cognitive tasks (Baddeley, 2012; Christophel et al., 2017; D’Esposito & Postle, 2015). It plays a fundamental role in various domains, including learning, problem-solving, decision-making, and attention regulation (Baddeley, 2012). Neurological and psychiatric disorders that affect working memory, such as Alzheimer’s disease, stroke, major depressive disorder, and obsessive-compulsive disorder, therefore have severe consequences for a person’s overall cognitive ability (D’Esposito & Postle, 2015). Consequently, understanding the factors that influence working memory performance has important implications for optimizing cognitive functioning and developing interventions to address working memory deficits.

Transcranial direct current stimulation (tDCS) has emerged as a promising non-invasive brain stimulation technique that modulates cortical excitability and neural activity (Polanía et al., 2018). By applying subthreshold electrical currents to specific brain regions through scalp electrodes, tDCS can selectively enhance or inhibit neuronal activity, leading to changes in brain function (Krause et al., 2017; Nitsche & Paulus, 2000, 2001; Sánchez-León et al., 2021). Consequently, a significant number of studies have applied tDCS to increase working memory performance. In particular, anodal tDCS to the left prefrontal cortex has been explored extensively (Wischnewski et al., 2021). Given the substantial number of studies, it is no surprise that different tDCS montages and intensities have been used to test effects on various tasks and outcome measures (Hill et al., 2016). Overall, meta-analytic evidence suggests that tDCS has a small modulatory effect on working memory (Brunoni & Vanderhasselt, 2014; Hill et al., 2016; Mancuso et al., 2016; Müller et al., 2022). However, different tDCS parameters can lead to vastly differing electric fields in the brain (Miranda et al., 2013; Opitz et al., 2015, 2018). As such, without taking montage variability into account, it is difficult to assess the effect of tDCS.

To address this issue, we recently developed a meta-analytic modeling approach that associates tDCS-related electric field distributions with behavioral effect sizes (Wischnewski et al., 2021). Electric field strengths are typically the largest between the anode and cathode. As a result, bilateral montages (e.g., F3-F4, F3-F8, F3-Fp2) will target the dorsolateral and medial prefrontal cortex. Extracephalic montages (e.g., F3-shoulder), on the other hand, result in more lateralized electric fields with the lower dorsolateral prefrontal and inferior frontal cortex as targets. With our method, the relative regional contributions of electric fields to the behavioral effects are accounted for and the brain regions that are associated with the largest tDCS-induced changes in working memory can be identified. With meta-modeling, we computed the electric field distributions of tDCS montages used across multiple studies that investigated working memory performance. For a given location in the brain—a node in the brain model—a specific electric field strength value is obtained. Subsequently, for each node, the electric field strength values across different studies are correlated with the observed behavioral effect sizes related to this stimulation montage. This results in a brain map that represents brain areas where tDCS-induced electric field values correlate, positively or negatively, with working memory performance. In other words, the map shows the most likely regions to be affected by tDCS if targeted, with larger electric fields relating to larger modulation in working memory.

Using this method, we demonstrated that prefrontal anodal tDCS has a positive effect on working memory when electric fields are largest in Brodmann areas (BA) 45 and 47. These results relate to general working memory effects, non-regarding working memory type, or the investigated outcome measure. However, imaging studies suggest that besides domain-general (Nee et al., 2013), there are also domain-specific function brain areas related to working memory (Kim et al., 2012; Lycke et al., 2008; Rottschy et al., 2012; Suchan, 2008). For example, verbal working memory is associated with activity in language areas, such as Broca’s area (BA 44 and 45) (Emch et al., 2019; Rottschy et al., 2012). In contrast, during visuospatial working memory, this area is not significantly involved, and activation is rather observed in other prefrontal (Dores et al., 2017; Kim et al., 2012; Li et al., 2022; Owen et al., 2005) and parietal regions (Dores et al., 2017; Wallentin et al., 2008). Additionally, investigation of speed and accuracy of performance can result in differing activation maps. Whereas maintenance and, consequently, accuracy relate to specific verbal and visuospatial working memory regions, reaction times are associated with regions that relate to general cognitive speed, such as the dorsolateral prefrontal cortex (Standage et al., 2014; van Veen et al., 2008).

With domain specificity in mind, we anticipated that the effects of tDCS will not be the same for all outcome measures and that electric fields in different areas will be associated with different working memory aspects. Using our previously established meta-modeling approach (Wischnewski et al., 2021), we compared the effects of tDCS-related electric fields between working memory accuracy and reaction time, verbal, and visuospatial working memory, and working memory performance during (online) or after (offline) stimulation. Our results show that the previously established region of BA 45/47 primarily relates to online verbal working memory accuracy. With these findings, we provide further insights into the functional areas that are causally involved in working memory tasks. Also, these results may be of guidance in the modulation of working memory by tDCS in neurological and psychiatric patient populations.

## 2 Materials and Methods

### 2.1 Study sample

Meta-modeling was performed on 354 outcome variables extracted from 67 peer-reviewed articles (PubMed & Google Scholar). The 58 studies on which we previously reported (Wischnewski et al., 2021) were included (Abellaneda-Pérez et al., 2020; Andrews et al., 2011; Baumert et al., 2020; Berryhill & Jones, 2012; Byrne et al., 2020; Carvalho et al., 2015; Cespón et al., 2017; Deldar et al., 2018, 2019; Di Rosa et al., 2019; Dumont et al., 2018; Faehling & Plewnia, 2016; Fregni et al., 2005; Friehs & Frings, 2019; Gill et al., 2015; Gladwin et al., 2012; Hill et al., 2017, 2018, 2019; Hoy et al., 2013; Hussey et al., 2015, 2020; Jeon & Han, 2012; Jones et al., 2015; Jongkees et al., 2019; Ke et al., 2019; Keeser et al., 2011; Koshy et al., 2020; Lally et al., 2013; Lukasik et al., 2018; Luque-Casado et al., 2019; Martin et al., 2013; Meiron & Lavidor, 2013; Moreno et al., 2015; Mulquiney et al., 2011; Murphy et al., 2020; Mylius et al., 2012; Naka et al., 2018; Nikolin et al., 2015, 2017, 2018, 2019; Nilsson et al., 2015; Ohn et al., 2008; Papazova et al., 2020; Pope et al., 2015; Rabipour et al., 2018; Ramaraju et al., 2020; Richmond et al., 2014; Röhner et al., 2018; Splittgerber et al., 2020; Talsma et al., 2017, 2018; Teo et al., 2011; Trumbo et al., 2016; Wang et al., 2019; Weintraub-Brevda & Chua, 2019; Zaehle et al., 2011). Nine additional studies were added to our database in March 2023 (Au et al., 2022; Karthikeyan et al., 2021; Maheux-Caron et al., 2021; Maldonado & Bernard, 2021; Martin et al., 2023; Teixeira-Santos et al., 2022; Voegtle et al., 2022; Zhao et al., 2022; Živanović et al., 2021). Studies were included based on the following inclusion criteria: I) Studies were published in a peer-reviewed journal. II) Full-text of the article was available in English. III) Reported data were collected in healthy volunteers (no restrictions on age or gender). IV) Studies included sham and/or baseline control measurements. V) At least one of the tDCS montages used in the article positioned the anode over the left prefrontal cortex. VI) At least one outcome measure investigated tDCS-related effects on working memory. VII) Studies presented results on working memory after a single session of tDCS. In the case of multi-session studies, only first-session results were used for analysis (Au et al., 2022; Ke et al., 2019; Lally et al., 2013; Martin et al., 2013; Richmond et al., 2014; Teixeira-Santos et al., 2022; Wang et al., 2019). VIII) Studies reported effect sizes, averages, and standard deviations in text or tables, or visual presentation of data in form of figures and supplementary data allowed for calculation of effect sizes, using WebPlotDigitizer (Rohatgi, 2022). A risk of bias assessment was performed, the results of which are presented in Supplementary Figure 1. Briefly, there were no concerns of a selection, attrition, and reporting bias. Blinding of personnel executing the experimental setup and analysis was unclear in the majority of studies. As the present study summarizes previous findings and does not include any experimental procedures, no ethical approval or informed consent was required.

The 354 outcome variables were gathered from various working memory tasks: N-back, Sternberg, Corsi block tapping, paced auditory serial addition and/or subtraction task, digit span, change detection task, internal shift task, delayed working memory task, and other customized working memory tasks. Note that in the context of the n-back task, we did not include 0-back and 1-back trials since they typically serve as control tasks and performance reaches a ceiling level. The main reported outcome measures were 1) accuracy, which included hit rate, percentage correct and sensitivity d (N = 181), and reaction time (N = 144). These outcome measures include effect sizes of any working memory type (verbal or visuospatial) and collected at any time (online or offline). Other outcome measures included maximum achieved n, which is the highest n in an n-back task, where participants still score above chance, forward span, and backward span. Due to the limited sample sizes, these were not further analyzed. 2) The tasks tested verbal (N = 271) and visuospatial (N = 73) working memory, regardless of performance measurement (accuracy or reaction time) and collected at any time (online or offline). 3) Working memory performance was assessed either during tDCS (online: N = 164) or immediately after tDCS (offline: N = 190). These outcome measures include effect sizes of any outcome measure (accuracy or reaction time) and working memory type (verbal or visuospatial).

### 2.2 Effect size calculation

The effect sizes we report here reflect the difference between the active and control conditions. Specifically, we calculated the effect size of working memory performance during left prefrontal anodal tDCS and subtracted the effect size of the sham and/or baseline condition. Using mean and standard deviation, Hedges’ *g* was computed, corresponding to Cohen’s *d* with a correction for inflation by studies with small sample sizes (Hedges & Olkin, 1985). We obtained Hedges’ *g* for all 354 outcome measures. When Hedges’ *g* is positive, it signifies improvement in working memory performance due to tDCS, compared to a control condition. Conversely, a negative Hedges’ *g* suggests a decline in working memory performance caused by tDCS, compared to a condition. $\bar{\text{G}}$ denotes cumulative effect size estimates.

### 2.3 Meta-modeling of working memory performance

All finite element modeling simulations were run using SimNIBS version 3.2. (Thielscher et al., 2015) and MATLAB 2020b. For each study included, we simulated the distribution of tDCS electric fields. The simulations utilized the precise tDCS montage, including intensity, as well as electrode location, size, shape, material, and orientation, as reported in each respective study. We used an individual head model of a healthy adult male (between 25-35 years old) and commonly used realistic conductivity values of different tissue types were applied: σ_skin_ = 0.465 S/m, σ_bone_ = 0.01 S/m, σ_cerebrospinal_ fluid = 1.654 S/m, σ_gray matter_ = 0.275 S/m, and σ_white matter_ = 0.126 S/m (Windhoff et al., 2013). All electric field simulations were run on the subject overlays, which consisted of 267,855 elements (nodes), meaning that there are the same number of electric field values (in mV/mm) for each tDCS montage. Further, to get a sense of PEC variability across gender and age, we performed the analyses on three additional head models (Supplementary Fig. 5): one younger (approximate age 25-35) female, one older (approximate age 65-75) male, and one older (approximate age 65-75) female. These head models were retrieved from the Human Connectome project (https://www.humanconnectome.org) and OpenNeuro (https://openneuro.org) databases. The same tissue conductivity values were used.

**Fig. 1.**
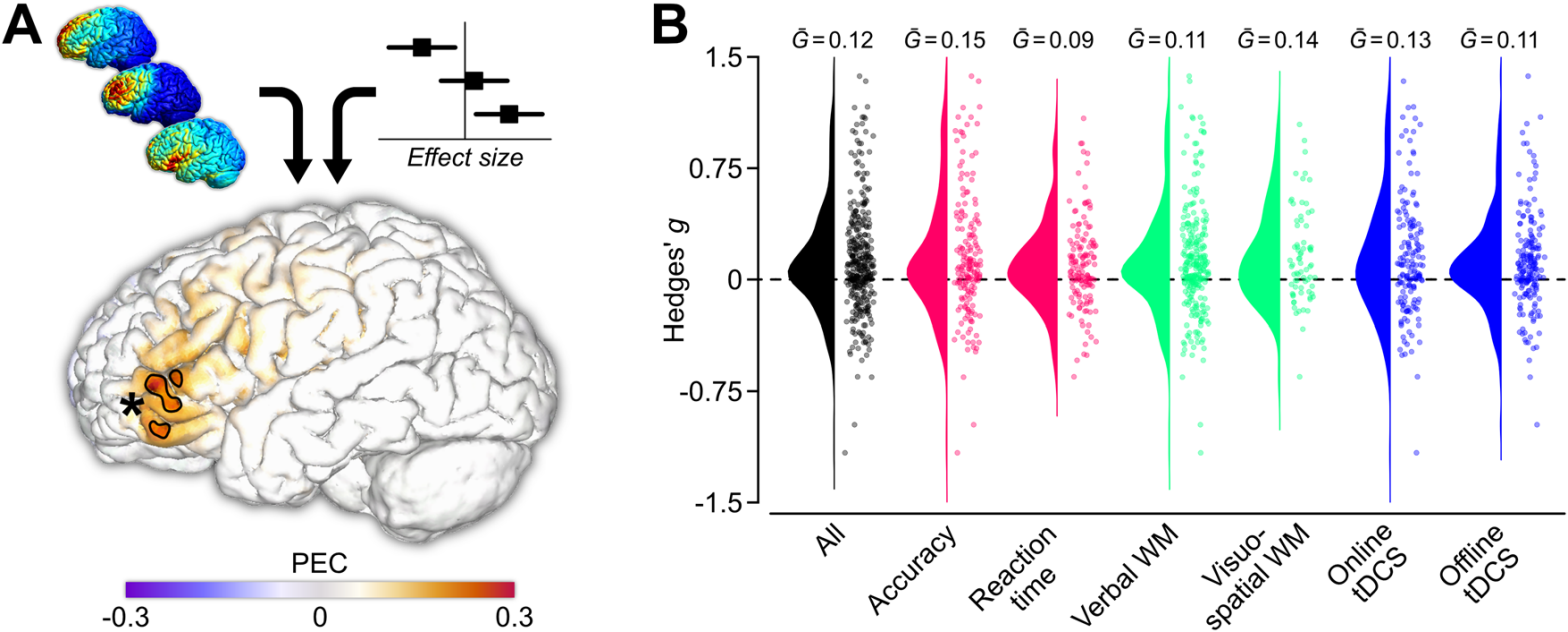
(A) Using a meta-modeling approach, we relate tDCS electric field distributions to behavioral effect size estimates. The resulting performance–electric field correlation (PEC) map shows which brain areas are associated with improved working memory performance when targeted by anodal tDCS. Previously, we have shown that Brodmann areas 45 and 47 are associated with domain-general working memory improvements (Wischnewski et al., 2021). *Indicates significant areas after permutation testing (p < 0.05) in the outlined areas. (B) Effect sizes (Hedges’ g) for all included studies and for each domain.

We used a previously established method to map brain regions where tDCS electric fields relate to improved working memory (Wischnewski et al., 2021) (Fig. 1A, Supplementary Fig. 2). For each node we calculated the correlation between electric field value and behavioral effect size, referred to as the performance-electric field correlation (PEC). Each PEC value represents the correlation between two vectors of the same length, namely 1) the electric field strengths per node across all studies and 2) the Hedges’ g across all studies. The vector length depends on the number of outcome measures. In total, 354 outcome measures were collected, of which a subset was chosen for six analyses (accuracy, reaction time, verbal working memory, visuospatial working memory, online assessment, offline assessment). Therefore, the full dataset consists of one 267,855 x 354 matrix of electric field strength values and one 1 x 354 vector of effect size values. All rows and a subset of columns (see section 2.1 for the sample size) were evaluated for each analysis. Each row of the electric field matrix was correlated with the effect size vector, resulting in a vector of 267,855 x 1 correlations. In other words, one correlation value for each node of the brain model, which allows for generating a map of PEC values. Regions where PEC > 0 relate to better working memory performance with increasing electric field strength. Regions where PEC < 0 relate to worsening working memory performance with increasing electric field strength.

Following the PEC calculation, we employed permutation testing to determine the significance of PEC. For this, we compared the actual PEC values to 1000 permutations of randomized PEC values, that is, a 267,855 x 1000 matrix. From these 1000 values per node, a distribution is generated. Subsequently, the position of the actual PEC values within this distribution is assessed, resulting in an estimate of significance (p-value) (Wischnewski et al., 2021). As the primary hypothesis of prefrontal anodal tDCS is to improve working memory, we utilized a one-sided distribution for our primary analysis. Two-sided p-values are presented in the Supplementary Data. Subsequently, we employed the Human Connectome Project multimodal parcellation atlas (HCP-MMP) to gain insight into the function of brain areas that contain significant PEC values (Glasser, Coalson, et al., 2016; Glasser, Smith, et al., 2016). The HCP-MMP contains 180 regions per hemisphere (360 regions in total). We identified significant brain regions when the 75^th^ percentile of nodes within a parcel was significant after permutation testing. For visualization, we used the ggseg library implemented in R (version 4.3), displaying the HCP-MMP parcels on an inflated brain surface (Mowinckel & Vidal-Piñeiro, 2020).

## 3 Results

### 3.1 Effect size estimates

We gathered 354 outcome measures from 67 peer-reviewed articles on the effects of left prefrontal anodal tDCS on working memory performance. Combining all outcomes resulted in an average effect size of $\bar{\text{G}}$ = 0.14. This effect is considered small (Cohen, 1977), and results were variable (Fig. 1B). Furthermore, average effect sizes were comparable for different performance measures (accuracy: $\bar{\text{G}}$ = 0.16, and reaction time: $\bar{\text{G}}$ = 0.10), working memory types (verbal: $\bar{\text{G}}$ = 0.13, and visuospatial: $\bar{\text{G}}$ = 0.15), and time of assessment (online: $\bar{\text{G}}$ = 0.16, and offline: $\bar{\text{G}}$ = 0.11).

### 3.2 PEC accuracy vs reaction time

**Fig. 2.**
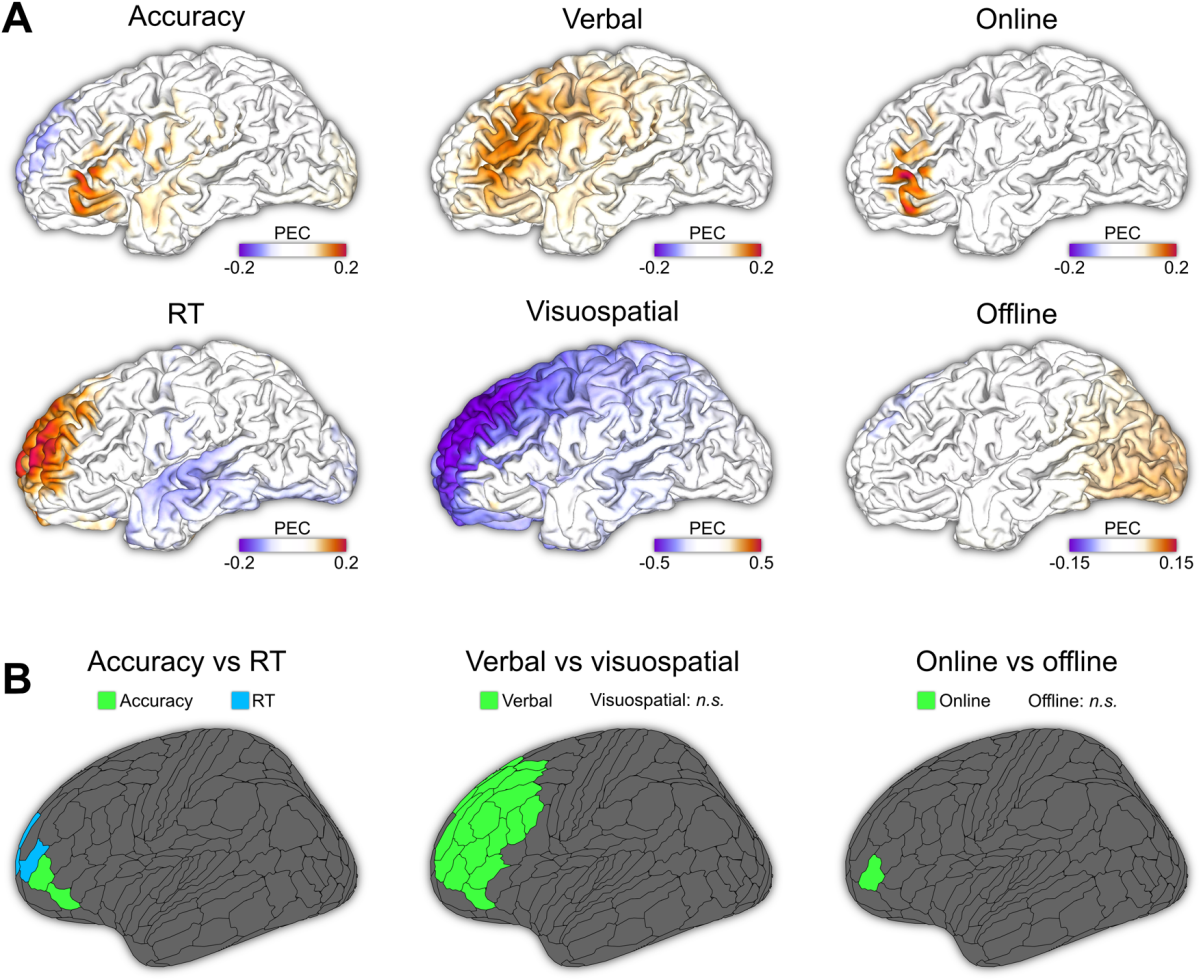
(A) Maps of PEC values for accuracy, reaction time, verbal and visuospatial working memory, online tDCS, and offline tDCS. (B) Brain regions (based on the HCP-MMP atlas) with significant PEC values.

For outcome measures on working memory accuracy, the robust maximum PEC = 0.173 (99.9^th^ percentile) was observed at MNI coordinates [-52.1, 37.5, 4,6] (Fig. 2A, left upper panel). Significant PEC values were located in the lateral BA 47 and posterior BA 47r of the left prefrontal cortex (Fig. 2B, left panel, Supplementary Fig. 3). For outcome measures on working memory reaction time, the robust maximum PEC = 0.201 was at MNI coordinates [-24.4, 60.1, 19.2] (Fig. 2A, left lower panel). PEC values reached significance in anterior BA 9, ventral border of BA 9-BA 46, and posterior BA 10p in the left prefrontal cortex (Fig. 2B, left panel; Supplementary Fig. 3), as well as anterior BA 9, middle BA 9, and dorsal BA 10 in the right prefrontal cortex.

### 3.3 PEC verbal vs visuospatial working memory

For verbal working memory, the robust maximum PEC = 0.155 was observed at MNI coordinates [-28.7, 13.7, 41.5] (Fig. 2A, middle upper panel). However, significant areas were stretched out over a large area of 26 parcels within the left prefrontal cortex (Fig. 2B, middle panel, Supplementary Fig. 3), including the anterior cingulate cortex (BA 24 and 32), inferior frontal junction (BA 8 and 44), inferior frontal gyrus (BA 45 and 47), and dorsolateral prefrontal cortex (BA 9 and 46). In contrast, for visuospatial working memory, no regions were related to significant working memory improvement (Fig. 2A, middle lower panel, Fig 2B, middle panel). Rather, significant negative PEC values were observed in the anterior, dorsolateral, and dorsomedial prefrontal regions (Supplementary Fig. 4).

### 3.4 PEC online vs. offline tDCS

Outcomes obtained during tDCS (online) were associated with a robust maximum PEC = 0.190, observed at MNI coordinates [-49.8, 40.6, 7.4] (Fig. 2B, right upper panel). Significant PEC values were located in the left posterior BA 46r (Fig. 2B, right panel, Supplementary Fig. 3). For offline tDCS, no significant PEC values were observed (Fig 2A, right lower panel, Fig. 2B, right panel).

### 3.5 PEC variability

To get a sense of the variability of PEC maps across age and gender, we performed the same analyses in three more head models: one healthy younger female, one healthy older male, and one healthy older female. The results are presented in Supplementary Figure 5. Although the precise PEC values and extent slightly differed between head models, generally the same spatial patterns were observed as in the main analysis.

## 4 Discussion

The goal of the present study was to identify brain regions that relate to improved working memory performance when targeted by anodal tDCS. To that end, we used a recently developed method of combining tDCS electric field simulations with meta-analytic behavioral effect size estimates (Wischnewski et al., 2021). Resulting maps demonstrate stimulation of which brain areas are most likely associated with improved working memory performance. First, we found that anodal tDCS improves accuracy on working memory when inferior frontal regions (BA 47) are stimulated, while reaction time during working memory assessment is related to stimulation of dorsolateral and anterior prefrontal areas (BA 9 and 10). Second, anodal tDCS to left prefrontal areas is associated with improved verbal working memory, whereas there is no improvement in visuospatial working memory. Third, improvement of working memory was observed for studies applying tDCS during a task (online), while assessment after stimulation (offline) was not associated with performance improvement.

The results of the present study expand on our previous results (Wischnewski et al., 2021). There, we found that domain general working memory performance is related to anodal tDCS electrics in the lower dorsolateral prefrontal and upper inferior frontal gyrus. Here, we show that these present results were likely driven by verbal tasks, that measured accuracy, using online tDCS. Besides sub-analyses on domain-specific working memory and measurement types, we expanded our meta-modeling method by implementing the results into the HCP-MMP atlas. Overall, with our work we hope to add insights into the causal involvement of brain regions in domain-specific working memory tasks. Furthermore, our results may guide tDCS placement for future experimental and clinical research with the goal of improving working memory.

Psychometric research has shown a positive association between working memory capacity and processing speed (Schmiedek et al., 2007; Wilhelm & Oberauer, 2006). Despite this link, we found that the effects of anodal tDCS on working memory accuracy and reaction time relate to distinct regions (Fig. 2A). In support of our observations, imaging studies have demonstrated a critical role of the dorsolateral prefrontal cortex for processing speed during working memory (Marshuetz et al., 2000; Takeuchi et al., 2012). In line, the dorsolateral prefrontal cortex has been proposed as a central region encoding speed-accuracy trade-offs (Standage et al., 2014; van Veen et al., 2008). Furthermore, Veltman and colleagues (Veltman et al., 2003) have shown that the left dorsolateral prefrontal cortex is associated with the response phase during a Sternberg and N-back task. Conversely, they showed that the encoding of working memory items is more strongly associated with inferior frontal gyrus activation (Veltman et al., 2003). This finding is in agreement with our results which also suggested tDCS electric fields at the inferior frontal gyrus (BA 47) relate to improved working memory accuracy. Other imaging studies have confirmed the role of inferior frontal activity in working memory encoding and maintenance, and thus accuracy (Chen & Desmond, 2005; Courtney et al., 1997; Li et al., 2022; Todd et al., 2011), although other parietal and prefrontal areas may be involved as well (Li et al., 2022; Rottschy et al., 2012).

When comparing PEC maps for different stimuli, we found that anodal tDCS positively affects verbal but not visuospatial working memory. Verbal working memory improvements were associated with electric fields in many prefrontal regions. Verbal information is encoded and maintained within the phonological loop (Baddeley, 2012). Besides the above-mentioned inferior frontal and dorsolateral areas, meta-analyses of imaging studies show that phonological rehearsal is also associated with language-related areas, such as BA 44 and 45 (Emch et al., 2019; Owen et al., 2005; Rottschy et al., 2012). In line with this, we found a significant association between verbal working memory performance and electric field strength in the inferior frontal junction (BA 44/45). These regions are often referred to as Broca’s area, which generally is associated with speech production, but are also involved in response inhibition (Papagno et al., 2017; Tomiyama et al., 2022). In future research, it would be interesting to disentangle the different functional contributions (speech initiation and inhibition) of the inferior frontal gyrus and how it relates to working memory. Further, note that here we only investigated the left hemisphere. While the inferior frontal gyrus is part of the phonological loop in the left hemisphere, the right inferior frontal gyrus is associated with the ventral attention and salience network (Allan et al., 2020; Cazzoli et al., 2021). Yet, the present sub-analyses on how tDCS to right hemispheric networks impacts working memory performance require more data, as the amount of studies using right prefrontal tDCS in working memory was too small (Wischnewski et al., 2021).

In contrast, prefrontal tDCS was not associated with visuospatial working memory improvement. Moreover, a negative association was observed in the dorsolateral and dorsomedial prefrontal regions when performing a two-sided analysis (Supplementary Fig. 4). The role of dorsal prefrontal regions in spatial working memory is a topic of ongoing debate. While Barbey and colleagues (Barbey et al., 2013) showed that lesions to the dorsolateral prefrontal cortex result in the worsening of verbal and spatial working memory, Mackey, Devinsky, Doyle, Meager, et al. (2016) found that lesions in this region have no impact on spatial working memory. Instead, precentral (BA 6 and 8) and inferior frontal gyrus activity (BA 47) may relate to visuospatial working memory (Dores et al., 2017; Li et al., 2022; Lycke et al., 2008; Mackey, Devinsky, Doyle, Meager, et al., 2016; Owen et al., 2005), as well as activation of a region in the parietal cortex (Curtis, 2006; Mackey, Devinsky, Doyle, Golfinos, et al., 2016; Owen et al., 2005). In this study, we did not find an association between electric fields in precentral areas and improved visuospatial working memory. For BA 47, a small area of positive PEC values was observed (Supplementary Fig. 3), which did not reach significance for the overall area. As such, whether inferior frontal gyrus stimulation improves visuospatial working memory remains inconclusive. However, our findings suggest that anodal tDCS to the dorsolateral and dorsomedial cortex does not positively, but rather negatively, impact working memory performance. Notably, the analysis on visuospatial working memory was performed on the smallest sample size (n=73). Furthermore, none of the included studies directly targeted the medial prefrontal cortex. As such, we can only speculate on why a reduction in performance was observed. Given that the brain has limited resources, activation of the prefrontal regions could possibly reduce engagement of other regions that are involved in the visuospatial working memory, such as parietal areas (Brem et al., 2014). However, this hypothesis needs to be tested in future research.

Finally, we compared studies that investigated working memory during the application of tDCS (online) or after tDCS (offline). Online anodal tDCS that targets the inferior frontal gyrus was associated with improved performance. For offline anodal tDCS, neither a positive nor a negative association was found in any region. When disregarding tDCS montages, the overall effect size of online studies ($\bar{\text{G}}$ = 0.13) was comparable to that of offline studies ($\bar{\text{G}}$ = 0.11). Our results do not imply that offline stimulation effects are ineffective per se, but rather that no clearly defined area could be attributed to these effects in this meta-analysis. For instance, some studies demonstrate superior effects after compared to during stimulation (Friehs & Frings, 2019; Živanović et al., 2021). Physiologically, the working hypothesis is that online anodal tDCS effects are associated with a small shift in resting membrane potential, which increases the likelihood of neural firing in the stimulated area. Offline effects are related to network-level plastic effects, similar to long-term potentiation (Stagg & Nitsche, 2011; Stagg et al., 2018). Arguably, online effects are, therefore, more regional than offline effects, which in our analysis is reflected by a more clearly defined region (Jamil & Nitsche, 2017) .

To get a sense of how variable PEC maps are across gender and age, we reran our analyses in three additional head models: one younger female and two older adults (male and female). Spatial patterns were consistent, meaning that electric field values correlated with working memory performance in the same regions in all head models. However, some slight variability in PEC magnitude and extent was observed. In part, this can be explained by variability in electric field distributions, which are a consequence of inter-individual differences in head and brain anatomy. Electric field magnitudes tend to reduce with age given the loss of grey matter volume in older individuals (Antonenko et al., 2021; McCann & Beltrachini, 2021). In contrast, the PEC reflects a measure of variance, meaning that the linearity of the relationship between electric fields and behavioral effect size determines these values, rather than the electric field magnitude per se. This explains why for some analyses, such as on accuracy and online tDCS, PEC values were larger in the head models of older volunteers. While it is beyond the scope of the present study, in future work it would be interesting to study the variability of brain anatomy related to gender and age on meta-modeling results in more detail.

While the present meta-analysis shows regional specific effects of electric fields, it should be noted that imaging studies have demonstrated the possibility of remote tDCS-related changes in metabolic activity. For instance, Abellaneda-Perez et al. (2020) found reduced activity throughout the default mode network after prefrontal (F3-Fp2) tDCS during working memory. Also, increased fronto-parietal connectivity has been reported after prefrontal tDCS (Claaß et al., 2023). To get a better understanding of how distributions of electric fields and metabolic activity relate, future research might compare working memory related fMRI maps and the current meta-modeling approach.

The clinical application of tDCS has grown in recent years to treat symptoms of neurological and psychiatric disorders (Fregni et al., 2021; Kuo et al., 2017; Razza et al., 2023; Salehinejad et al., 2020). Various studies have shown the beneficial effects of tDCS in depression (Razza et al., 2023), attention-deficit hyperactivity disorder (Salehinejad et al., 2020), and stroke (Yan et al., 2020). Since various diseases are associated with deficits in executive functions, including working memory, the present results can guide tDCS montages for therapeutic use. Notably, with significant maximum PEC values between 0.155 for verbal working memory and 0.201 for reaction time measurements, the explained variance ranges between 2.5 and 4% and thus should be considered as a small-to-moderate effect size. These subtle effects are not superior to other methods, such as working memory training (Karbach & Verhaeghen, 2014) or neurofeedback (Yeh et al., 2022). However, it should be noted that the present study involved studies in healthy volunteers that applied a single session of tDCS. Recently, meta-analytic evidence suggested that multi-session tDCS has small beneficial effects on cognitive performance for a median duration of 1 month (Pergher et al., 2022). Further, beneficial effects on working memory have been meta-analytically demonstrated in patients with schizophrenia (Liu et al., 2021). In future work, it would be fascinating to investigate the effects of tDCS on working memory in neuropsychiatric populations using meta-modeling. Additionally, other behavioral domains could be considered, as well as meta-modeling with other types of non-invasive brain stimulation, such as transcranial magnetic stimulation and transcranial alternating current stimulation.

## Supplementary Material

Supplementary Material

## Data Availability

Data are extracted from peer-reviewed articles that are available on PubMED and Google Scholar. Codes used in this study are openly available, provided by SimNIBS (https://simnibs.github.io/simnibs/build/html/index.html). In-house codes to perform the meta-modeling analysis are openly available on GitHub (https://github.com/Miles2708/Metamodeling_WM.git).
