# Supplementary material for: Meta-modeling the effects of anodal left prefrontal transcranial direct current stimulation on working memory performance
Source: Imaging Neurosci (Camb). 2024 Jan 25;2:imag-2-00078. doi: 10.1162/imag_a_00078 (PMC12224430; doi:10.1162/imag_a_00078)
Supplement: Supplementary Material [file imag_a_00078-supp.pdf]

Meta-modeling the effects of anodal left prefrontal transcranial direct current stimulation on working memory performance

Miles Wischnewski, Taylor A. Berger, Alexander Opitz

Supplementary materials

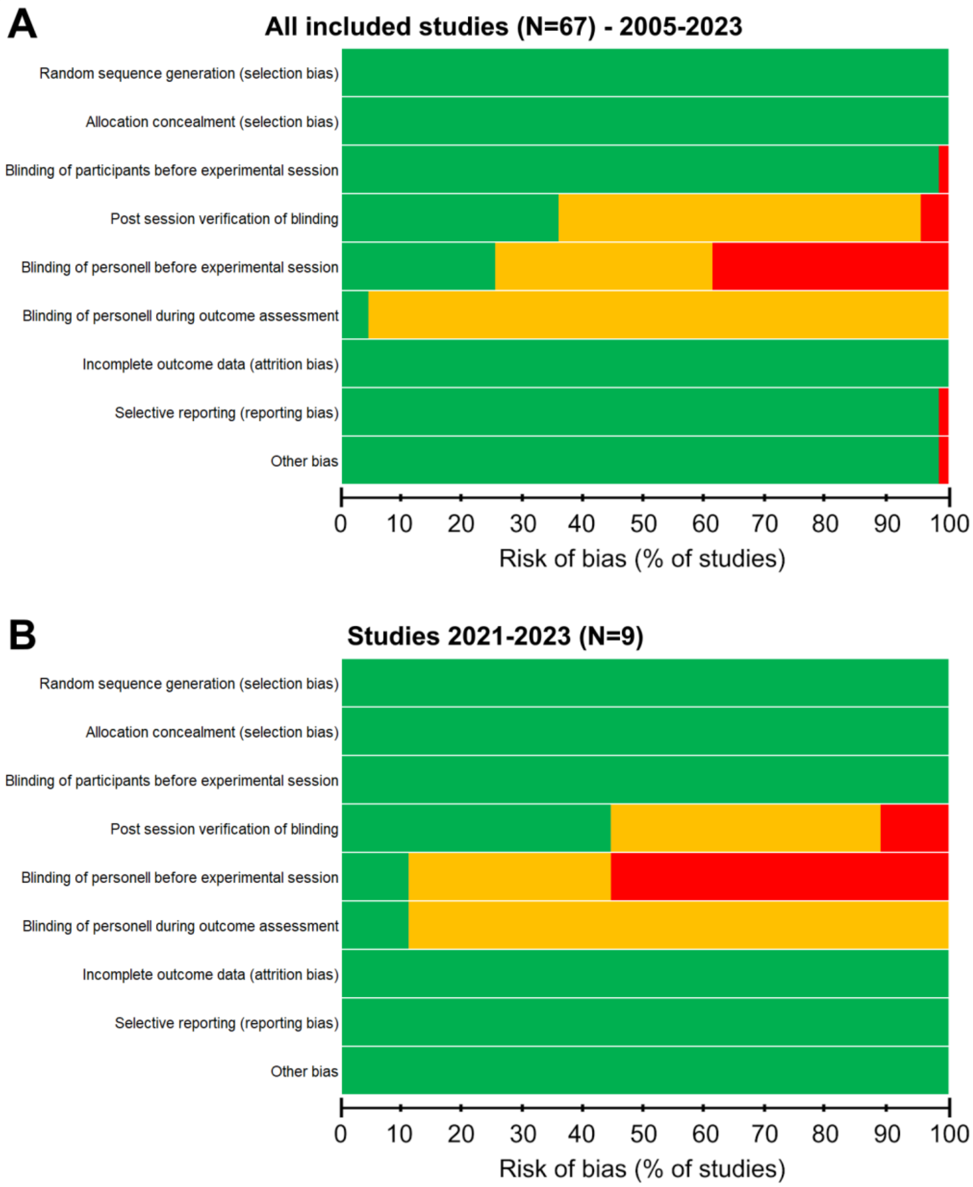

Supplementary Figure 1. Risk of bias analysis of A) all included studies (N=67). Note that 58 out of the 67 studies were already included in our previous publication (Wischnewski et al., 2021). Therefore, we also show in B) a risk of bias analysis of the newly added N=9 papers. It can be concluded that the risk of selection, attrition and reporting bias is low. Whereas almost all studies initially blinded participants, the success of blinding was assessed in less than half of the studies. This is important as active tDCS may cause different sensations compared to sham tDCS. Future studies should therefore employ questionnaires assessing successful blinding after stimulation. In a large number of studies, the personnel performing stimulation was either unblinded, or it was unreported whether they were blinded. Furthermore, the vast majority of studies did not report whether data analysis was performed blindly. Based on this assessment, we urge future investigations to report on the blinding of personnel that perform experiments and assess the data.

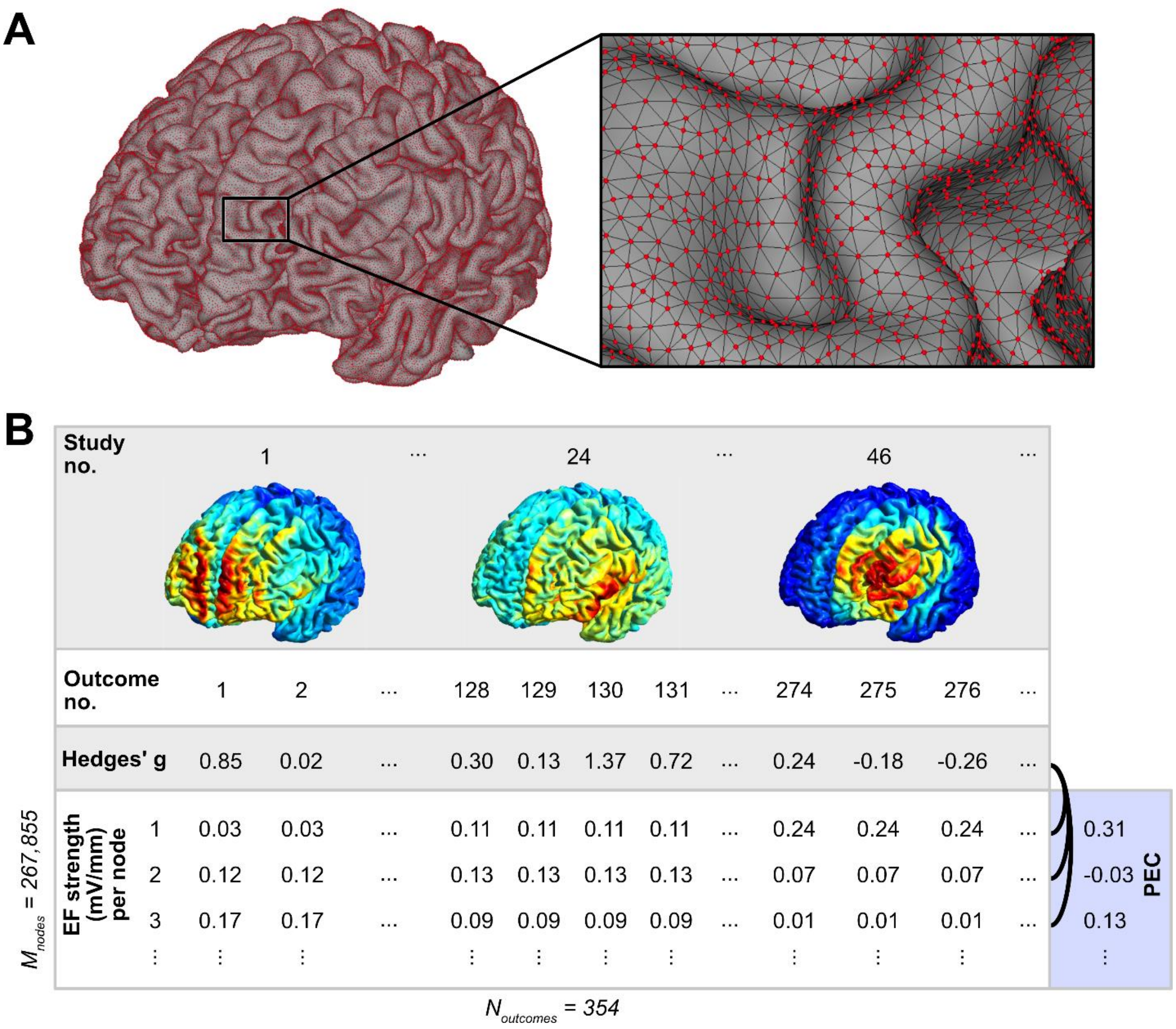

Supplementary Figure 2. Calculation of PEC values. A) The mesh of the grey matter surface consists of a large amount of nodes. Specifically, the head model used for the main analysis consisted of 267,855 grey matter nodes. For each node the PEC value was calculated. B) All together 354 outcome measures were obtained. Note that for each of the six sub-analysis (accuracy, reaction time, verbal working memory, visuospatial working memory, online assessment, offline assessment) only a subset of these outcomes measures was used. The sample sizes for each analysis are reported in the main text. For each study electric field distributions were calculated. This means that for each node in each study an electric field (EF) strength value is obtained. The results is a 267,855x354 matrix of all electric field values across all studies. Additionally, for each outcome measure the behavioral effect size, expressed in Hedges' g is obtained, resulting in a 1x354 vector. Subsequently, the Hedges' g vector is correlated with each row of the electric field matrix. From this a 267,855x1 vector of PEC values is obtained. These values are then implemented in the head model which gives us the PEC value map.

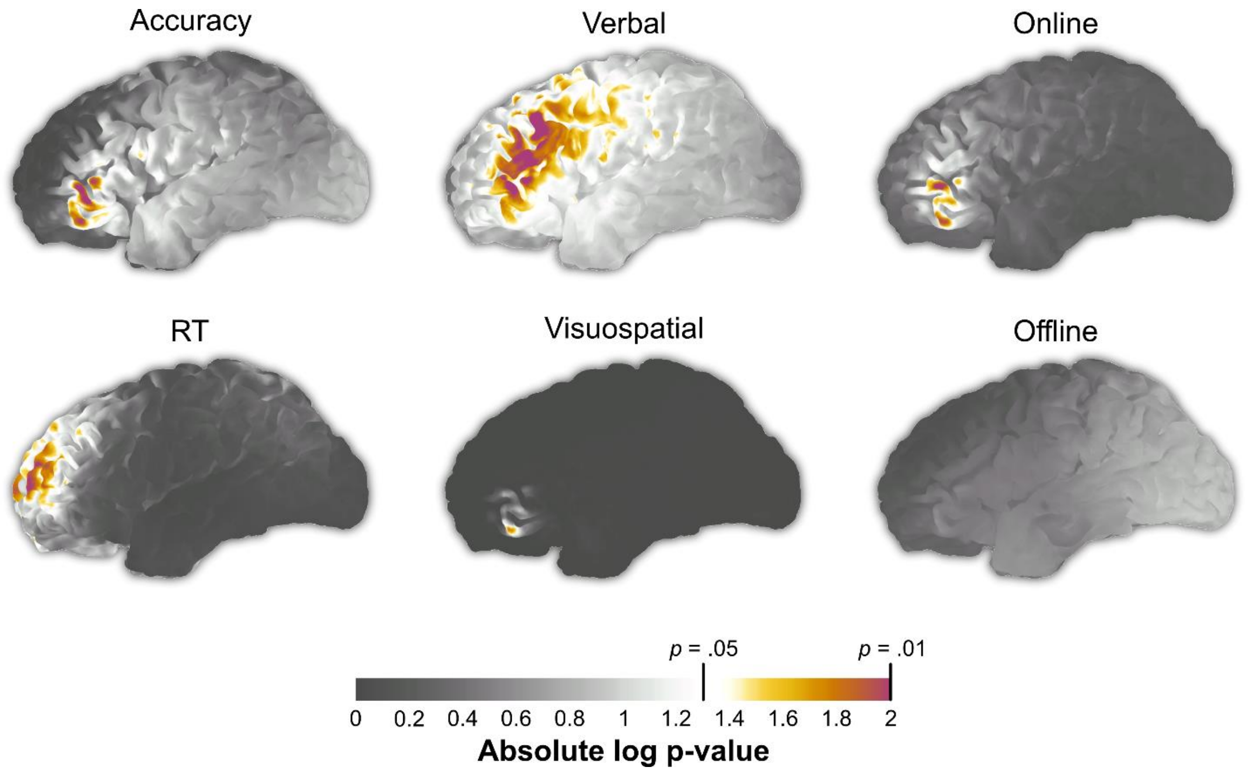

Supplementary Figure 3. Distribution of p-values after one-sided permutation testing for positive PEC values. For visualization we show the absolute log of the p-values, where values > 1.301 correspond to p < 0.05.

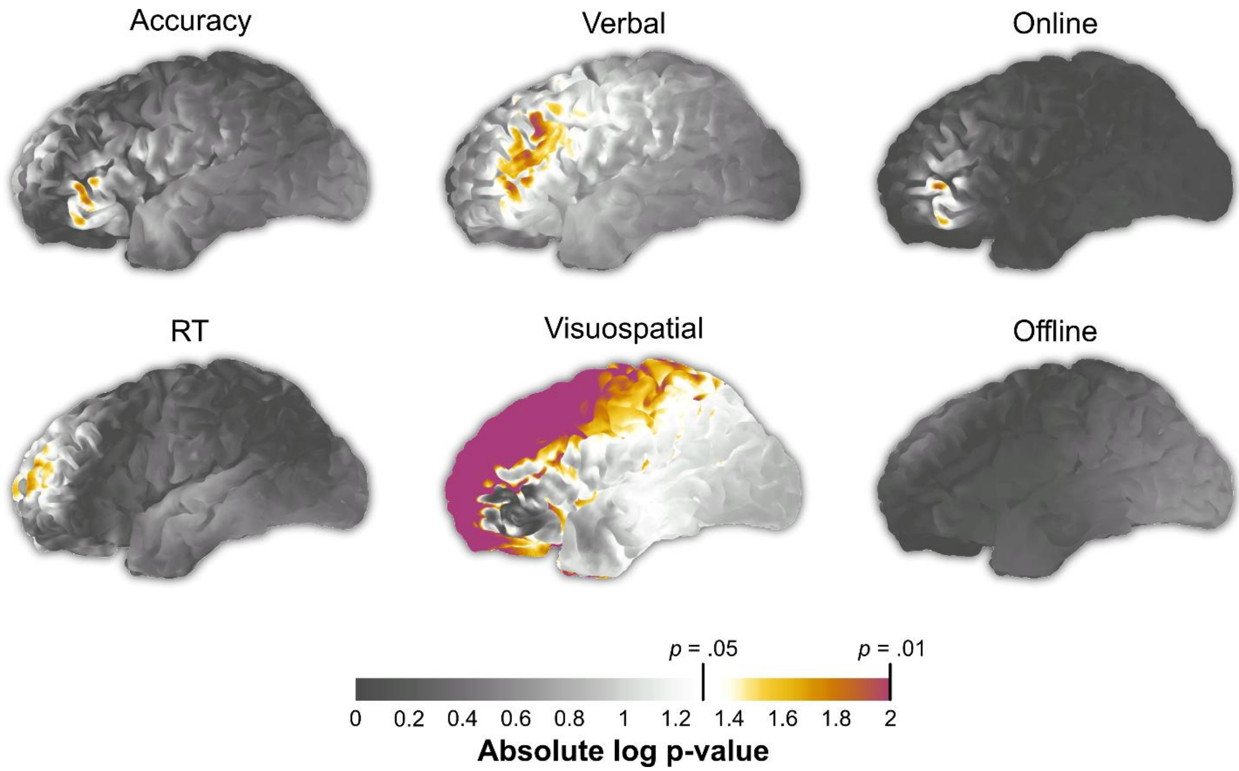

Supplementary Figure 4. Distribution of p-values after two-sided permutation testing for positive and negative PEC values. For visualization we show the absolute log of the p-values, where values > 1.301 correspond to p < 0.05.

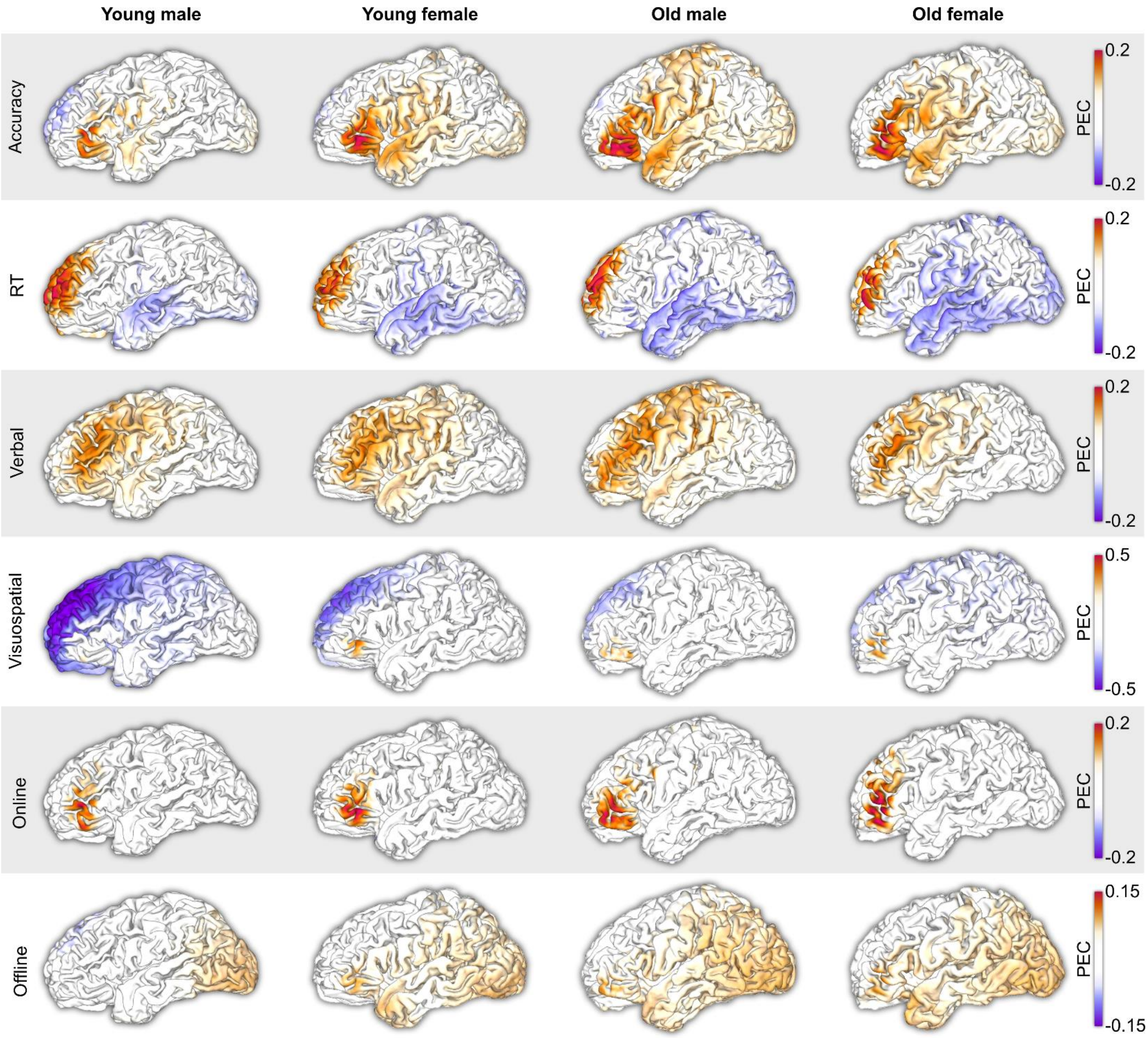

Supplementary Figure 5. PEC maps for the different working memory types and measurements for four head models. The first column represents the results on a young healthy male (approx. age 25-35), which is also presented in the main manuscript. Additionally, the second, third and fourth column show the results on a younger female (approx. age 25-35), older male (approx. age 65-75), and older female (approx. age 65-75) respectively. These head models were retrieved from the Human Connectome project (https://www.humanconnectome.org) and OpenNeuro (https://openneuro.org) databases. When comparing the maps slight variability can be observed in the magnitude and extent of the PEC values. However, the spatial patterns are consistent across head models, suggesting that electric fields of the same regions correlate to working memory performance.

Table S1. Overview of included studies.

Study no.	Reference	Study no.	Reference	Study no.	Reference	Study no.	Reference
1	Fregni et al., 2005	18	Gill et al., 2015	35	Naka et al., 2018	52	Byrne et al., 2020
2	Ohn et al., 2008	19	Hussey et al., 2015	36	Nikolin et al., 2018	53	Hussey et al., 2020
3	Andrews et al., 2011	20	Jones et al., 2015	37	Rabipour et al., 2018	54	Koshy et al., 2020
4	Keeser et al., 2011	21	Moreno et al., 2015	38	Rohner et al., 2018	55	Murphy et al., 2020
5	Mulquiney et al., 2011	22	Nikolin et al., 2015	39	Talsma et al., 2018	56	Papazova et al., 2020
6	Theo et al., 2011	23	Nilsson et al., 2015	40	Baumert et al., 2019	57	Ramaraju et al., 2020
7	Zaehle et al., 2011	24	Pope et al., 2015	41	Deldar et al., 2019	58	Splittberger et al., 2020
8	Berryhill et al., 2012	25	Faehling et al., 2016	42	Di Rosa et al., 2019	59	Karthikeyan et al., 2021
9	Gladwin et al., 2012	26	Trumbo et al., 2016	43	Friehs & Frings, 2019	60	Maheux-Caron et al., 2021
10	Jeon et al., 2012	27	Cespon et al., 2017	44	Hill et al., 2019	61	Maldonado & Bernard, 2021
11	Mylius et al., 2012	28	Hill et al., 2017	45	Jongkees et al., 2019	62	Zivanovic et al., 2021
12	Hoy et al., 2013	29	Nikolin et al., 2017	46	Ke et al., 2019[a]	63	Au et al., 2022[a]
13	Lally et al., 2013[a]	30	Talsma et al., 2017	47	Luque-Casado et al. 2019	64	Teixeira-Santos et al., 2022[a]
14	Martin et al., 2013[a]	31	Deldar et al., 2018	48	Nikolin et al., 2019	65	Voegtle et al., 2022[a]
15	Meiron et al., 2013	32	Dumont et al., 2018	49	Wang et al., 2019[a]	66	Zhao et al., 2022
16	Richmond et al., 2014[a]	33	Hill et al., 2018	50	Weintraub-Brevda & Chua, 2019	67	Martin et al., 2023
17	Carvalho et al., 2015	34	Lukasik et al., 2018	51	Abellaneda-Perez et al., 2020		

[a] Multi-day experiments. Only results from the first day were used here.

Table S2. Montages and parameters, as well as tasks, outcome measures and effect sizes per included study

Study no.	Anode (size in cm²)	Cathode (size in cm²)	Intensity (mA)	Outcome no.	Task	Outcome	WM type	Online Offline	Hedges' g
1	F3 (35)	rSOR (35)	1	1	3-back	Accuracy	Verb	On	0.85
				2	3-back	RT	Verb	On	0.02
2	F3 (25)	rSOR (25)	1	3	3-back (M1)	Accuracy	Verb	On	-0.05
				4	3-back (M1)	RT	Verb	On	0.62
				5	3-back (M2)	Accuracy	Verb	On	0.28
				6	3-back (M2)	RT	Verb	On	1.09
				7	3-back (M3)	Accuracy	Verb	On	0.57
				8	3-back (M3)	RT	Verb	On	0.85
				9	3-back (M4)	Accuracy	Verb	Off	0.35
				10	3-back (M4)	RT	Verb	Off	0.86
3	F3 (35)	rSOR (35)	1	11	DSF	Span	Verb	Off	-0.36
				12	DSB	Span	Verb	Off	0.04
				13	DSF (+n-back dur. tDCS)	Span	Verb	Off	0.39
				14	DSB (+n-back dur. tDCS)	Span	Verb	Off	0.25
4	F3 (35)	rSOR (35)	2	15	2-back	Accuracy	Verb	Off	0.42
				16	2-back	RT	Verb	Off	0.29
5	F3 (35)	rSOR (35)	1	17	2-back	Accuracy	VS	Off	-0.20
				18	2-back	RT	VS	Off	0.42
6	F3 (35)	rSOR (35)	1	19	Sternberg	Accuracy	Verb	Off	-0.03
				20	Sternberg	RT	Verb	Off	0.00

				21	3-back (M1)	Accuracy	Verb	On	0.10
				22	3-back (M1)	RT	Verb	On	0.37
				23	3-back (M2)	Accuracy	Verb	On	-0.02
				24	3-back (M2)	RT	Verb	On	0.15
			2	25	Sternberg	Accuracy	Verb	Off	0.21
				26	Sternberg	RT	Verb	Off	0.12
				27	3-back (M1)	Accuracy	Verb	On	-0.06
				28	3-back (M1)	RT	Verb	On	0.10
				29	3-back (M2)	Accuracy	Verb	On	0.03
				30	3-back (M2)	RT	Verb	On	0.36
7	F3 (35)	IMST (35)	1	31	2-back	Accuracy	Verb	Off	0.38
				32	2-back	RT	Verb	Off	-0.38
8	F3 (35)	EC (35)	1.5	33	2-back (verbal)	Accuracy	Verb	Off	0.38
				34	2-back (verbal)	Accuracy	Verb	Off	0.07
				35	2-back (spatial)	Accuracy	VS	Off	0.21
				36	2-back (spatial)	Accuracy	VS	Off	-0.23
9	F3 (35)	rSOR (35)	1	37	Sternberg no interference, easy	Accuracy	Verb	On	-0.18
				38	Sternberg no interference, easy	RT	Verb	On	0.18
				39	Sternberg no interference, med	Accuracy	Verb	On	0.19
				40	Sternberg no interference, med	RT	Verb	On	0.02
				41	Sternberg no interference, hard	Accuracy	Verb	On	0.25
				42	Sternberg no interference, hard	RT	Verb	On	0.01
				43	Sternberg with interference, easy	Accuracy	Verb	On	-0.12
				44	Sternberg with interference, easy	RT	Verb	On	0.31
				45	Sternberg with interference, med	Accuracy	Verb	On	0.34
				46	Sternberg with interference, med	RT	Verb	On	0.35
				47	Sternberg with interference, hard	Accuracy	Verb	On	0.59
				48	Sternberg with interference, hard	RT	Verb	On	0.48
10	F3 (35)	rSOR (35)	1	49	DSF	Span	Verb	Off	0.09
				50	DSB	Span	Verb	Off	0.11
11	F3 (35)	rSOR (35)	2	51	2-back	Accuracy	Verb	On	0.59
				52	2-back	RT	Verb	On	0.10
12	F3 (35)	rSOR (35)	1	53	2-back (M1)	Accuracy	Verb	Off	0.51
				54	2-back (M1)	RT	Verb	Off	0.26
				55	2-back (M2)	Accuracy	Verb	Off	0.43
				56	2-back (M2)	RT	Verb	Off	0.16
				57	2-back (M3)	Accuracy	Verb	Off	-0.06
				58	2-back (M3)	RT	Verb	Off	0.46
				59	3-back (M1)	Accuracy	Verb	Off	-0.25
				60	3-back (M1)	RT	Verb	Off	0.02
				61	3-back (M2)	Accuracy	Verb	Off	0.13
				62	3-back (M2)	RT	Verb	Off	0.23
				63	3-back (M3)	Accuracy	Verb	Off	0.06
				64	3-back (M3)	RT	Verb	Off	0.22
			2	65	2-back (M1)	Accuracy	Verb	Off	0.45
				66	2-back (M1)	RT	Verb	Off	0.01
				67	2-back (M2)	Accuracy	Verb	Off	0.30

				68	2-back (M2)	RT	Verb	Off	0.00
				69	2-back (M3)	Accuracy	Verb	Off	0.23
				70	2-back (M3)	RT	Verb	Off	0.38
				71	3-back (M1)	Accuracy	Verb	Off	0.07
				72	3-back (M1)	RT	Verb	Off	-0.08
				73	3-back (M2)	Accuracy	Verb	Off	0.07
				74	3-back (M2)	RT	Verb	Off	0.16
				75	3-back (M3)	RT	Verb	Off	0.16
				76	3-back (M3)	Accuracy	Verb	Off	0.28
13	F3 (35)	EC (35)	1	77	3-back	Accuracy	Verb	On	1.00
				78	3-back	RT	Verb	On	0.03
				79	3-back	Accuracy	Verb	Off	0.07
				80	3-back	RT	Verb	Off	-0.02
14	F3 (35)	EC (100)	2	81	n-back[a]	Achieved N	Verb	On	0.30
15	F3-AF3 (16)	Cz (35)	2	82	2-back (male participants)	Accuracy	Verb	On	2.38
				83	2-back (female participants)	Accuracy	Verb	On	-0.28
16	F3 (35)	F4 (35)	1.5	84	Operation span	Span	Verb	On	0.21
				85	Symmetry span	Span	VS	On	0.36
17	F3 (35)	rSOR (35)	1	86	3-back	Accuracy	VS	On	0.71
18	F3 (25)	rSOR (25)	2	87	3-back	Accuracy	Verb	On	0.10
				88	PASAT (1-back dur. tDCS)	Accuracy	Verb	Off	-0.42
				89	PASAT (3-back dur. tDCS)	Accuracy	Verb	Off	0.99
19	F3 (1.3)	O1 (1.3)	2	90	2-back	Discriminability	Verb	On	-0.17
				91	2-back (with distractors)	Discriminability	Verb	On	-0.04
				92	4-back	Discriminability	Verb	On	0.32
				93	4-back (with distractors)	Discriminability	Verb	On	0.38
20	F3-F7 (35)	EC (35)	1.5	94	CDT active rehearsal (low perf.)	Accuracy	VS	Off	-0.06
				95	CDT active rehearsal (high perf.)	Accuracy	VS	Off	0.68
				96	CDT passive rehearsal (low perf.)	Accuracy	VS	Off	0.43
				97	CDT passive rehearsal (high perf.)	Accuracy	VS	Off	-0.09
				98	CDT low incentive (low perf.)	Accuracy	VS	Off	0.03
				99	CDT low incentive (high perf.)	Accuracy	VS	Off	0.03
				100	CDT high incentive (low perf.)	Accuracy	VS	Off	0.14
				101	CDT high incentive (high perf.)	Accuracy	VS	Off	0.67
21	F3 (25)	F4 (25)	2	102	2-back	Accuracy	Verb	Off	0.79
				103	2-back	RT	Verb	Off	0.92
				104	IST (non-emotional)	Switch cost	VS	Off	-0.36
				105	IST (emotional)	Switch cost	VS	Off	-0.27
22	F3 (3.14)	HD (3.14)	2	106	3-back	Accuracy	Verb	Off	0.10
				107	3-back	RT	Verb	Off	0.17
23	F3 (35)	rSOR (100)	1	108	3-back (M1)	Accuracy	VS	On	0.10
				109	3-back (M1)	RT	VS	On	0.25
				110	3-back (M2)	Accuracy	VS	On	0.22
				111	3-back (M2)	RT	VS	On	-0.15
				112	3-back (M3)	Accuracy	VS	On	0.20
				113	3-back (M3)	RT	VS	On	-0.03
				114	3-back (M1)	Accuracy	VS	Off	0.21

				115	3-back (M1)	RT	VS	Off	0.09
				116	3-back (M2)	Accuracy	VS	Off	0.20
				117	3-back (M2)	RT	VS	Off	-0.10
			2	118	3-back (M1)	Accuracy	VS	On	0.01
				119	3-back (M1)	RT	VS	On	0.20
				120	3-back (M2)	Accuracy	VS	On	0.23
				121	3-back (M2)	RT	VS	On	-0.05
				122	3-back (M3)	Accuracy	VS	On	-0.21
				123	3-back (M3)	RT	VS	On	-0.08
				124	3-back (M1)	Accuracy	VS	Off	-0.10
				125	3-back (M1)	RT	VS	Off	0.07
				126	3-back (M2)	Accuracy	VS	Off	0.01
				127	3-back (M2)	RT	VS	Off	-0.11
24	F3 (25)	EC (25)	2	128	PASAT	Accuracy	Verb	Off	0.30
				129	PASAT	RT	Verb	Off	0.13
				130	PASST	Accuracy	Verb	Off	1.37
				131	PASST	RT	Verb	Off	0.72
25	F3 (35)	EC (35)	0.5	132	DWMT (neutral)	Accuracy	Verb	On	-0.08
				133	DWMT (neutral)	RT	Verb	On	-0.51
				134	DWMT (emotional)	Accuracy	Verb	On	-0.30
				135	DWMT (emotional)	RT	Verb	On	-0.42
			1	136	DWMT (neutral)	Accuracy	Verb	On	0.00
				137	DWMT (neutral)	RT	Verb	On	-0.02
				138	DWMT (emotional)	Accuracy	Verb	On	-0.16
				139	DWMT (emotional)	RT	Verb	On	-0.14
			1.5	140	DWMT (neutral)	Accuracy	Verb	On	-0.27
				141	DWMT (neutral)	RT	Verb	On	-0.35
				142	DWMT (emotional)	Accuracy	Verb	On	-0.38
				143	DWMT (emotional)	RT	Verb	On	-0.15
26	F3 (25)	EC (25)	2	144	Spat. 3-back (M1)	Accuracy	VS	On	0.05
				145	Spat. 3-back (M2)	Accuracy	VS	On	0.04
				146	Spat. 3-back (M3)	Accuracy	VS	On	-0.13
				147	Spat. 3-back (M4)	Accuracy	VS	On	-0.21
				148	Spat. 3-back + spat. tr.	Accuracy	VS	Off	-0.33
				149	Spat. 3-back (M1)	RT	VS	On	0.51
				150	Spat. 3-back (M2)	RT	VS	On	0.00
				151	Spat. 3-back (M3)	RT	VS	On	-0.21
				152	Spat. 3-back (M4)	RT	VS	On	-0.65
				153	Spat. 3-back + spat. tr.	RT	VS	Off	-0.12
				154	Verb. 3-back + spat. tr.	Accuracy	Verb	Off	-0.98
	F3 (25)	EC (25)	2	155	Verb. 3-back (M1)	Accuracy	Verb	On	1.34
				156	Verb. 3-back (M2)	Accuracy	Verb	On	0.97
				157	Verb. 3-back (M3)	Accuracy	Verb	On	1.16
				158	Verb. 3-back (M4)	Accuracy	Verb	On	1.00
				159	Verb. 3-back + verb. tr.	Accuracy	Verb	Off	1.10
				160	Verb. 3-back (M1)	RT	Verb	On	0.02
				161	Verb. 3-back (M2)	RT	Verb	On	0.03

				162	Verb. 3-back (M3)	RT	Verb	On	-0.31
				163	Verb. 3-back (M4)	RT	Verb	On	0.09
				164	Verb. 3-back + verb. tr.	RT	Verb	Off	-0.25
				165	Spat. 3-back + verb. tr.	Accuracy	VS	Off	0.11
27	F3 (16)	EC (50)	1.5	166	3-back (young participants)	Accuracy	Verb	Off	-0.12
				167	3-back (young participants)	RT	Verb	Off	-0.01
				168	2-back (old participants)	Accuracy	Verb	Off	0.26
				169	2-back (old participants)	RT	Verb	Off	0.00
28	F3 (12.6)	rSOR (12.6)	1	170	2-back (M1)	Accuracy	Verb	Off	-0.36
				171	2-back (M1)	RT	Verb	Off	0.14
				172	2-back (M2)	Accuracy	Verb	Off	0.07
				173	2-back (M2)	RT	Verb	Off	0.24
	F3 (3.14)	HD (3.14)	1	174	2-back (M1)	Accuracy	Verb	Off	-0.45
				175	2-back (M1)	RT	Verb	Off	0.23
				176	2-back (M2)	Accuracy	Verb	Off	0.01
				177	2-back (M2)	RT	Verb	Off	-0.04
29	F3 (16)	F4 (16)	2	178	3-back	Accuracy	Verb	On	-1.17
				179	3-back	RT	Verb	On	0.37
				180	3-back	Accuracy	Verb	Off	-0.66
				181	3-back	RT	Verb	Off	-0.24
30	F3 (35)	rSOR (35)	1	182	n-back[a]	Accuracy	Verb	On	0.12
				183	n-back[a] (M1)	Accuracy	Verb	Off	0.77
				184	n-back[a] (M2)	Accuracy	Verb	Off	0.71
31	F3 (35)	EC (35)	2	185	2-back	Accuracy	VS	On	0.09
				186	2-back	RT	VS	On	-0.05
				187	2-back + pain	Accuracy	VS	On	0.40
				188	2-back + pain	RT	VS	On	0.36
32	F3 (35)	rSOR (35)	1.5	189	NIH-examiner	WM score	VS	Off	-0.10
33	F3 (3.14)	HD (3.14)	1.5	190	2-back (M1)	Accuracy	Verb	Off	0.13
				191	2-back (M1)	RT	Verb	Off	0.15
				192	2-back (M2)	Accuracy	Verb	Off	-0.02
				193	2-back (M2)	RT	Verb	Off	0.12
	F3/P3 (3.14)	HD (3.14)	1.5	194	2-back (M1)	Accuracy	Verb	Off	0.03
				195	2-back (M1)	RT	Verb	Off	0.52
				196	2-back (M2)	Accuracy	Verb	Off	0.06
				197	2-back (M2)	RT	Verb	Off	0.46
	F3 (3.14)	HD (3.14)	1.5	198	3-back (M1)	Accuracy	Verb	Off	-0.24
				199	3-back (M1)	RT	Verb	Off	0.11
				200	3-back (M2)	Accuracy	Verb	Off	-0.04
				201	3-back (M2)	RT	Verb	Off	0.16
	F3/P3 (3.14)	HD (3.14)	1.5	202	3-back (M1)	Accuracy	Verb	Off	-0.03
				203	3-back (M1)	RT	Verb	Off	0.12
				204	3-back (M2)	Accuracy	Verb	Off	0.10
				205	3-back (M2)	RT	Verb	Off	0.15
34	F7 (48)	rSOR (30)	1.5	206	3-back	Accuracy	Verb	On	-0.02
				207	3-back	RT	Verb	On	0.05
				208	3-back	Accuracy	Verb	Off	0.00

				209	3-back	RT	Verb	Off	0.05
35	F3 (4)	HD (4)	1.5	210	Visual 3-back	Accuracy	Verb	On	1.09
				211	Visual 3-back	RT	Verb	On	0.78
				212	Auditory 3-back	Accuracy	Verb	On	0.06
				213	Auditory 3-back	RT	Verb	On	-0.28
				214	Visual 3-back	Accuracy	Verb	Off	1.05
				215	Visual 3-back	RT	Verb	Off	0.92
				216	Auditory 3-back	Accuracy	Verb	Off	-0.08
				217	Auditory 3-back	RT	Verb	Off	-0.16
36	F3 (16)	F4 (16)	1	218	3-back	Accuracy	Verb	On	0.12
				219	3-back	RT	Verb	On	0.15
				220	3-back	Accuracy	Verb	Off	0.51
				221	3-back	RT	Verb	Off	-0.04
			2	222	3-back	Accuracy	Verb	On	-0.47
				223	3-back	RT	Verb	On	0.16
				224	3-back	Accuracy	Verb	Off	-0.40
				225	3-back	RT	Verb	Off	0.04
37	F3 (35)	rSOR (35)	2	226	3-back low expectation	Accuracy	Verb + VS	On	-0.48
				227	3-back low expectation	False alarm	Verb + VS	On	-0.21
				228	3-back high expectation	Accuracy	Verb + VS	On	1.16
				229	3-back high expectation	False alarm	Verb + VS	On	0.43
38	F3 (35)	EC (35)	1	230	2-back	Accuracy	Verb	On	0.10
				231	2-back	RT	Verb	On	-0.01
				232	2-back	Accuracy	Verb	Off	0.22
				233	2-back	RT	Verb	Off	0.01
39	F3 (35)	rSOR (35)	1	234	n-back[a]	Accuracy	Verb	On	-0.42
				235	n-back[a]	RT	Verb	On	-0.50
				236	n-back[a]	Accuracy	Verb	Off	-0.42
				237	n-back[a]	RT	Verb	Off	-0.54
40	F3 (35)	EC (35)	1	238	1-, 2-, 3-back	Accuracy	Verb	Off	0.83
				239	1-, 2-, 3-back	RT	Verb	Off	0.25
41	F3 (35)	EC (35)	2	240	2-back	Accuracy	VS	On	0.51
				241	2-back	RT	VS	On	0.53
				242	2-back + pain	Accuracy	VS	On	0.46
				243	2-back + pain	RT	VS	On	0.52
42	F3-F7 (35)	EC (35)	1.5	244	Custom WM task + low reward	Accuracy	Verb	On	-0.19
				245	Custom WM task + low reward	RT	Verb	On	0.11
				246	Custom WM task + high reward	Accuracy	Verb	On	0.08
				247	Custom WM task + high reward	RT	Verb	On	0.27
				248	Custom WM task + no reward	Accuracy	Verb	Off	0.19
				249	Custom WM task + no reward	RT	Verb	Off	0.16
43	F3 (9)	EC	0.5	250	3-back	Accuracy	Verb	On	-0.20
				251	3-back	RT	Verb	On	-0.15
				252	3-back	Accuracy	Verb	Off	0.44
				253	3-back	RT	Verb	Off	0.53
44	F3 (3.14)	HD (3.14)	1.5	254	2-back	Accuracy	Verb	Off	0.30
				255	2-back	RT	Verb	Off	0.01

				256	2-back + PASAT dur. tDCS	Accuracy	Verb	Off	0.61
				257	2-back + PASAT dur. tDCS	RT	Verb	Off	0.03
				258	3-back	Accuracy	Verb	Off	0.10
				259	3-back	RT	Verb	Off	0.01
				260	3-back + PASAT dur. tDCS	Accuracy	Verb	Off	-0.02
				261	3-back + PASAT dur. tDCS	RT	Verb	Off	-0.01
45	F3 (35)	F4 (35)	1	262	2-back (met/met participants)	Accuracy	Verb	Off	-0.35
				263	2-back (met/met participants)	RT	Verb	Off	0.02
				264	4-back (met/met participants)	Accuracy	Verb	Off	0.16
				265	4-back (met/met participants)	RT	Verb	Off	-0.21
				266	2-back (val/met participants)	Accuracy	Verb	Off	-0.07
				267	2-back (val/met participants)	RT	Verb	Off	0.00
				268	4-back (val/met participants)	Accuracy	Verb	Off	-0.18
				269	4-back (val/met participants)	RT	Verb	Off	-0.19
				270	2-back (val/val participants)	Accuracy	Verb	Off	0.14
				271	2-back (val/val participants)	RT	Verb	Off	0.06
				272	4-back (val/val participants)	Accuracy	Verb	Off	-0.28
				273	4-back (val/val participants)	RT	Verb	Off	0.15
46	F3 (4.91)	HD (4.91)	1.5	274	n-back[a]	Achieved N	Verb	On	0.24
				275	3-back	Accuracy	Verb	On	-0.18
				276	4-back	Accuracy	Verb	On	-0.26
47	F3 (35)	rSOR (35)	1.5	277	DSB (M1)	Span	Verb	Off	0.16
				278	DSB (M2)	Span	Verb	Off	0.09
48	F3 (3.14)	HD (3.14)	2	279	3-back	Accuracy	Verb	On	0.12
				280	3-back	RT	Verb	On	0.01
				281	Dual 3-back	Accuracy	Verb	On	0.40
49	F3 (3.14)	HD (3.14)	2	282	n-back[a]	Achieved N	Verb	On	0.37
50	F7 (3.14)	HD (3.14)	2	283	DWMT easy (neutral)	Accuracy	VS	On	0.94
				284	DWMT easy (emotional)	Accuracy	VS	On	1.04
				285	DWMT hard (neutral)	Accuracy	VS	On	0.86
				286	DWMT hard (emotional)	Accuracy	VS	On	0.72
51	F3 (35)	rSOR (35)	2	287	2-back task	Accuracy	Verb	On	0.02
				288	2-back task	RT	Verb	On	0.50
				289	2-back task	Accuracy	Verb	Off	-0.13
				290	2-back task	RT	Verb	Off	0.26
				291	3-back task	Accuracy	Verb	On	0.17
				292	3-back task	RT	Verb	On	0.22
				293	3-back task	Accuracy	Verb	Off	-0.10
				294	3-back task	RT	Verb	Off	0.33
52	F3 (25)	rSOR (25)	1	295	DSB	Span	Verb	On	0.15
53	F3 (5*2)	EC (5*2)	2	296	2-back + exercise	Discriminability	Verb	On	0.41
				297	2-back	Discriminability	Verb	On	0.61
				298	4-back + exercise	Discriminability	Verb	On	0.40
				299	4-back	Discriminability	Verb	On	-0.04
54	F3 (3.14)	HD (3.14)	2	300	Custom WM task	Accuracy	Verb	Off	0.01
				301	Custom WM task	RT	Verb	Off	0.00
55	F3 (35)	rSOR (35)	1	302	Sternberg (M1)	Accuracy	Verb	Off	-0.46

				303	Sternberg (M1)	RT	Verb	Off	-0.17
				304	Sternberg (M2)	Accuracy	Verb	Off	-0.24
				305	Sternberg (M2)	RT	Verb	Off	-0.10
56	F3 (35)	EC (35)	1	306	2-back	Accuracy	Verb	On	0.02
				307	2-back	RT	Verb	On	0.03
				308	3-back	Accuracy	Verb	On	0.01
				309	3-back	RT	Verb	On	-0.22
	F3 (35)	EC (35)	2	310	2-back	Accuracy	Verb	On	0.02
				311	2-back	RT	Verb	On	-0.03
				312	3-back	Accuracy	Verb	On	0.02
				313	3-back	RT	Verb	On	0.10
57	F3 (35)	rSOR (35)	1.5	314	Verb. 2-back	Accuracy	Verb	Off	0.34
				315	VS. 2-back	Accuracy	VS	Off	0.54
58	F3 (25)	rSOR (25)	1	316	2-back	Accuracy	VS	On	0.18
				317	2-back	RT	VS	On	-0.19
				318	2-back	Accuracy	VS	Off	0.13
				319	2-back	RT	VS	Off	-0.01
	AF3/AF7/F3 (3.14)	Fp2/T7 (3.14)	1	320	2-back	Accuracy	VS	On	0.14
				321	2-back	RT	VS	On	-0.16
				322	2-back	Accuracy	VS	Off	0.17
				323	2-back	RT	VS	Off	-0.12
59	F3 (35)	rSOR (35)	1	324	2-back	Accuracy	VS	On	0.45
				325	2-back	Accuracy	VS	Off	0.40
60	F3 (3.14)	rSOR (35)	2	326	2-, 3-back	Accuracy	Verb + VS	On	0.00
61	F3-F5 (3.14)	HD (3.14)	2	327	Sternberg easy	Accuracy	Verb	Off	-0.02
				328	Sternberg easy	RT	Verb	Off	0.12
				329	Sternberg med	Accuracy	Verb	Off	0.01
				330	Sternberg med	RT	Verb	Off	0.07
				331	Sternberg hard	Accuracy	Verb	Off	-0.01
				332	Sternberg hard	RT	Verb	Off	-0.05
62	F3 (25)	EC (25)	1.5	333	Verb. 3-back	Accuracy	Verb	On	-0.22
				334	Verb. 3-back	RT	Verb	On	-0.31
				335	Spat. 3-back	Accuracy	VS	On	0.02
				336	Spat. 3-back	RT	VS	On	0.22
			1.8	337	Verb. 3-back	Accuracy	Verb	Off	0.09
				338	Verb. 3-back	RT	Verb	Off	0.26
				339	Spat. 3-back	Accuracy	VS	Off	-0.01
				340	Spat. 3-back	RT	VS	Off	-0.01
63	F3 (35)	rSOR (35)	2	341	n-back[a]	Achieved N	Verb	On	0.00
64	F3 (35)	rSOR (35)	2	342	n-back[a]	Achieved N	Verb + VS	On	0.46
65	F3 (35)	EC (35)	1	343	2-back	Accuracy	Verb	On	-0.39
				344	2-back	RT	Verb	On	-0.43
				345	2-back	Accuracy	Verb	Off	0.04
				346	2-back	RT	Verb	Off	-0.21
66	F3 (25)	rSOR (25)	1	347	Verb. 3-back	Accuracy	Verb	Off	0.00
				348	Verb. 3-back	RT	Verb	Off	0.20
				349	Spat. 3-back	Accuracy	VS	Off	0.10

				350	Spat. 3-back	RT	VS	Off	0.22
67	F3 (35)	F8 (35)	2	351	2-back	Accuracy	Verb	On	0.34
				352	2-back	RT	Verb	On	-0.12
	F3 (35)	EC (35)	2	353	2-back	Accuracy	Verb	On	0.00
				354	2-back	RT	Verb	On	0.24

[a]The n-level of the task was adaptively adjusted based on participants performance.

Abbreviations: Change detection task (CDT), delayed working memory task (DWMT), digit span backward (DSB), digit span forward (DSF), during (dur), extracephalic (EC), high-definition (HD), internal shift task (IST), left mastoid (lMST), medium (med), measurement (M), offline tDCS (off), online tDCS (on), paced auditory serial addition test (PASAT), paced auditory serial subtraction test (PASST), reaction time (RT), right supraorbital region (rSOR), spatial (spat), training (tr), verbal working memory (verb), visuospatial working memory (VS), working memory (WM).
